# Mechanisms and Modulation of Oxidative/Nitrative Stress in Type 4 Cardio-Renal Syndrome and Renal Sarcopenia

**DOI:** 10.3389/fphys.2018.01648

**Published:** 2018-11-26

**Authors:** Márta Sárközy, Zsuzsanna Z. A. Kovács, Mónika G. Kovács, Renáta Gáspár, Gergő Szűcs, László Dux

**Affiliations:** Department of Biochemistry, Faculty of Medicine, University of Szeged, Szeged, Hungary

**Keywords:** end-stage renal disease, uremic cardiomyopathy, uremic myopathy, inflammation, anti-oxidants, renin-angiotensin-aldosterone system (RAAS), exercise training, microRNA (miR)

## Abstract

Chronic kidney disease (CKD) is a public health problem and a recognized risk factor for cardiovascular diseases (CVD). CKD could amplify the progression of chronic heart failure leading to the development of type 4 cardio-renal syndrome (T4CRS). The severity and persistence of heart failure are strongly associated with mortality risk in T4CRS. CKD is also a catabolic state leading to renal sarcopenia which is characterized by the loss of skeletal muscle strength and physical function. Renal sarcopenia also promotes the development of CVD and increases the mortality in CKD patients. In turn, heart failure developed in T4CRS could result in chronic muscle hypoperfusion and metabolic disturbances leading to or aggravating the renal sarcopenia. The interplay of multiple factors (e.g., comorbidities, over-activated renin-angiotensin-aldosterone system [RAAS], sympathetic nervous system [SNS], oxidative/nitrative stress, inflammation, etc.) may result in the progression of T4CRS and renal sarcopenia. Among these factors, oxidative/nitrative stress plays a crucial role in the complex pathomechanism and interrelationship between T4CRS and renal sarcopenia. In the heart and skeletal muscle, mitochondria, nicotinamide adenine dinucleotide phosphate (NADPH) oxidases, uncoupled nitric oxide synthase (NOS) and xanthine oxidase are major ROS sources producing superoxide anion (O2^·−^) and/or hydrogen peroxide (H_2_O_2_). O2^·−^ reacts with nitric oxide (NO) forming peroxynitrite (ONOO^−^) which is a highly reactive nitrogen species (RNS). High levels of ROS/RNS cause lipid peroxidation, DNA damage, interacts with both DNA repair enzymes and transcription factors, leads to the oxidation/nitration of key proteins involved in contractility, calcium handling, metabolism, antioxidant defense mechanisms, etc. It also activates the inflammatory response, stress signals inducing cardiac hypertrophy, fibrosis, or cell death via different mechanisms (e.g., apoptosis, necrosis) and dysregulates autophagy. Therefore, the thorough understanding of the mechanisms which lead to perturbations in oxidative/nitrative metabolism and its relationship with pro-inflammatory, hypertrophic, fibrotic, cell death and other pathways would help to develop strategies to counteract systemic and tissue oxidative/nitrative stress in T4CRS and renal sarcopenia. In this review, we also focus on the effects of some well-known and novel pharmaceuticals, nutraceuticals, and physical exercise on cardiac and skeletal muscle oxidative/nitrative stress in T4CRS and renal sarcopenia.

## Introduction

### Clinical significance and classification of CKD

The prevalence of CKD varies between 7 and 12% in the general population worldwide and increases with age affecting more than 30% of people over 65 years (Walker et al., 2013; Musso et al., 2015; Romagnani et al., 2017). CKD is defined as the abnormal renal structure and/or dysfunction (glomerular filtration rate [GFR] <60 mL/min/1.73 m^2^) present for at least 3 months (KDIGO Work Group, 2013; Pinheiro da Silva and Vaz da Silva, 2016). It can be classified into five stages based on GFR values. G1 stage refers to normal (GFR >90 mL/min/1.73 m^2^), G2 to mildly decreased (GFR = 60–89 mL/min/1.73 m^2^), G3 to moderately decreased (GFR = 30–59 mL/min/1.73 m^2^), G4 to severely decreased kidney function (GFR = 15–29 mL/min/1.73 m^2^) and G5 to kidney failure including end-stage renal disease (ESRD) (GFR <15 mL/min/1.73 m^2^) which requires kidney replacement therapy for survival (KDIGO Work Group, 2013; Pinheiro da Silva and Vaz da Silva, 2016). Large population studies have demonstrated that all stages of CKD increase the risk of premature death mainly from CVD (Go et al., 2004; Kumar et al., 2014). CKD and ESRD patients have a 5- to 10-fold higher risk for developing CVD compared to age-matched controls (Duni et al., 2017). Despite the broad availability of medications to control the underlying diseases (e.g., hypertension, diabetes mellitus, hyperlipidemia, etc.) and renal replacement therapy, cardiovascular morbidity and mortality remain major challenges in the management of CKD patients.

### T4CRS

**Figure 1:**
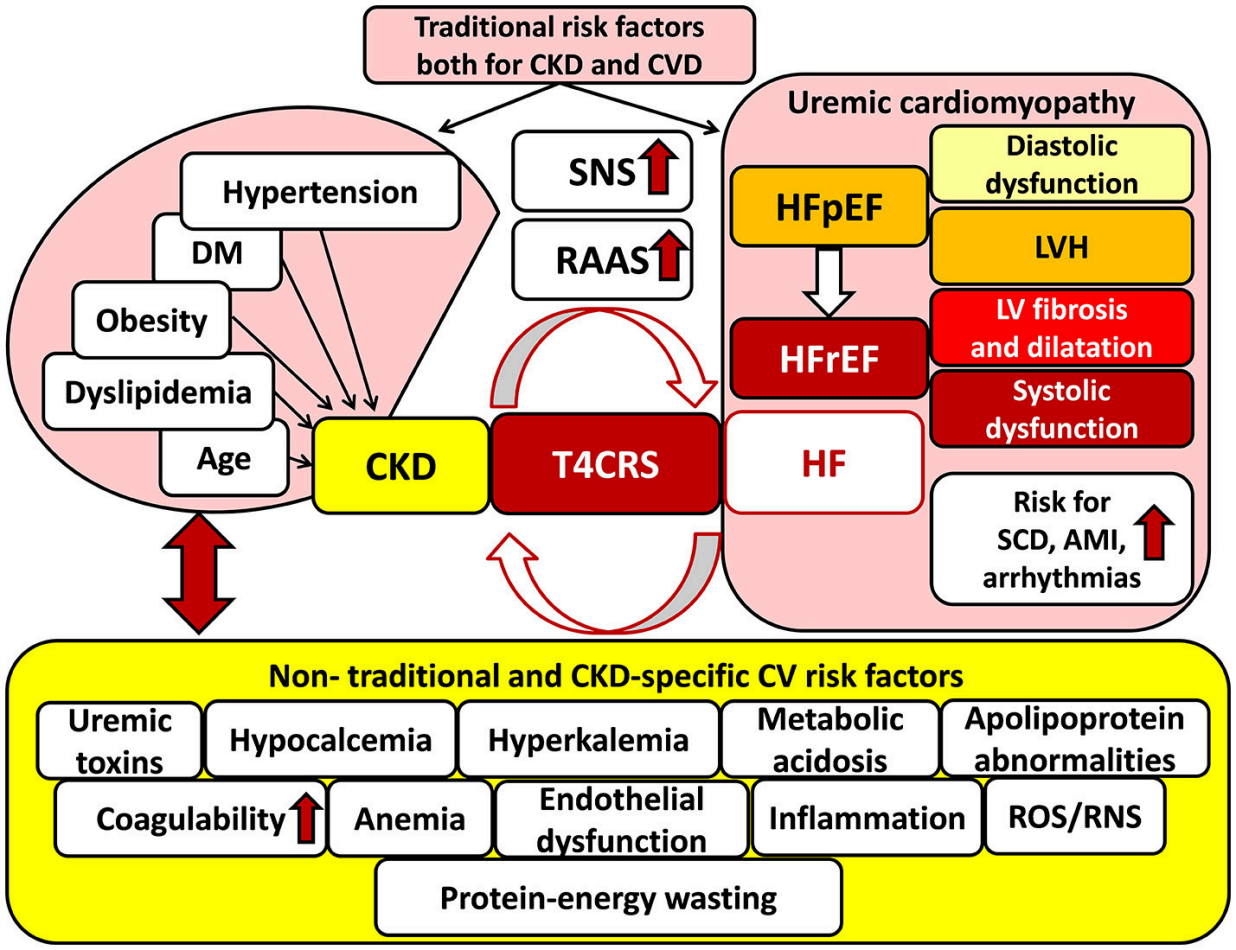
Traditional and non-traditional cardiovascular risk factors for T4CRS. AMI, acute myocardial infarction; CKD, chronic kidney disease; CVD, cardiovascular disease; DM, diabetes mellitus; HF, heart failure; HFpEF, heart failure with preserved ejection fraction; HFrEF, heart failure with reduced ejection fraction, LVH: left ventricular hypertrophy; RAAS, renin–angiotensin–aldosterone system; RNS, reactive nitrogen species; ROS, reactive oxygen species; SCD, sudden cardiac death, SNS: sympathetic nervous system; T4CRS, type 4 cardio-renal syndrome.

Recognition that CVD is a major cause of mortality in CKD patients, and conversely, deterioration in kidney function is associated with a poorer prognosis in CVD patients, led to the introduction of the term of cardio-renal syndrome defined by Ronco et al. (2008). The bidirectional interaction of renal and heart failure is the key concept in cardio-renal syndrome (Ronco et al., 2008; Kumar et al., 2014) (Figure 1). Dysfunction of each organ can induce and perpetuate injury in the other via complex hemodynamic, neurohormonal (e.g., over-activated SRS, RAAS, etc.), biochemical (e.g., increased oxidative/nitrative stress, etc.), inflammatory, and immunologic pathways (Bock and Gottlieb, 2010; Brisco and Testani, 2014) (Figure 1). The cardio-renal syndrome has five subtypes which classification is based on the organ affected primarily (heart, kidney or both, e.g., in sepsis) and the time frame (acute or chronic) (see for reviews: Ronco et al., 2008; Husain-Syed et al., 2015). T4CRS is the subtype in which CKD is the primary disease including, e.g., diabetic, hypertensive, autoimmune, etc. nephropathy forms, and induces the progression of chronic heart failure with preserved ejection fraction (HFpEF) and later heart failure with reduced ejection fraction (HFrEF) (Husain-Syed et al., 2015; Pinheiro da Silva and Vaz da Silva, 2016) (Figure 1). The CKD-associated chronic functional, structural and electrophysiological changes of the heart are also called uremic cardiomyopathy (Husain-Syed et al., 2015). It is characterized by left ventricular hypertrophy (LVH) and diastolic dysfunction in the HFpEF phase and later interstitial fibrosis, capillary rarefication, and systolic dysfunction in the HFrEF phase (Kocsis et al., 2012; Husain-Syed et al., 2015) (Figure 1). Epidemiological and imaging studies supposed that the leading manifestation of uremic cardiomyopathy is LVH (Mark et al., 2006; Siedlecki and Muslin, 2008; Alhaj et al., 2013). The prevalence of LVH significantly increases with the progression of CKD (31, 50, and 90% in stages G3, G4, and G5, respectively) (Cerasola et al., 2011; Duni et al., 2017). The severity and persistence of LVH are strongly correlated with acute cardiovascular events (e.g., sudden cardiac death, arrhythmias, and acute myocardial infarction) and mortality risk in T4CRS (Zoccali et al., 2004; Husain-Syed et al., 2015; Duni et al., 2017) (Figure 1).

### Renal sarcopenia

**Figure 2:**
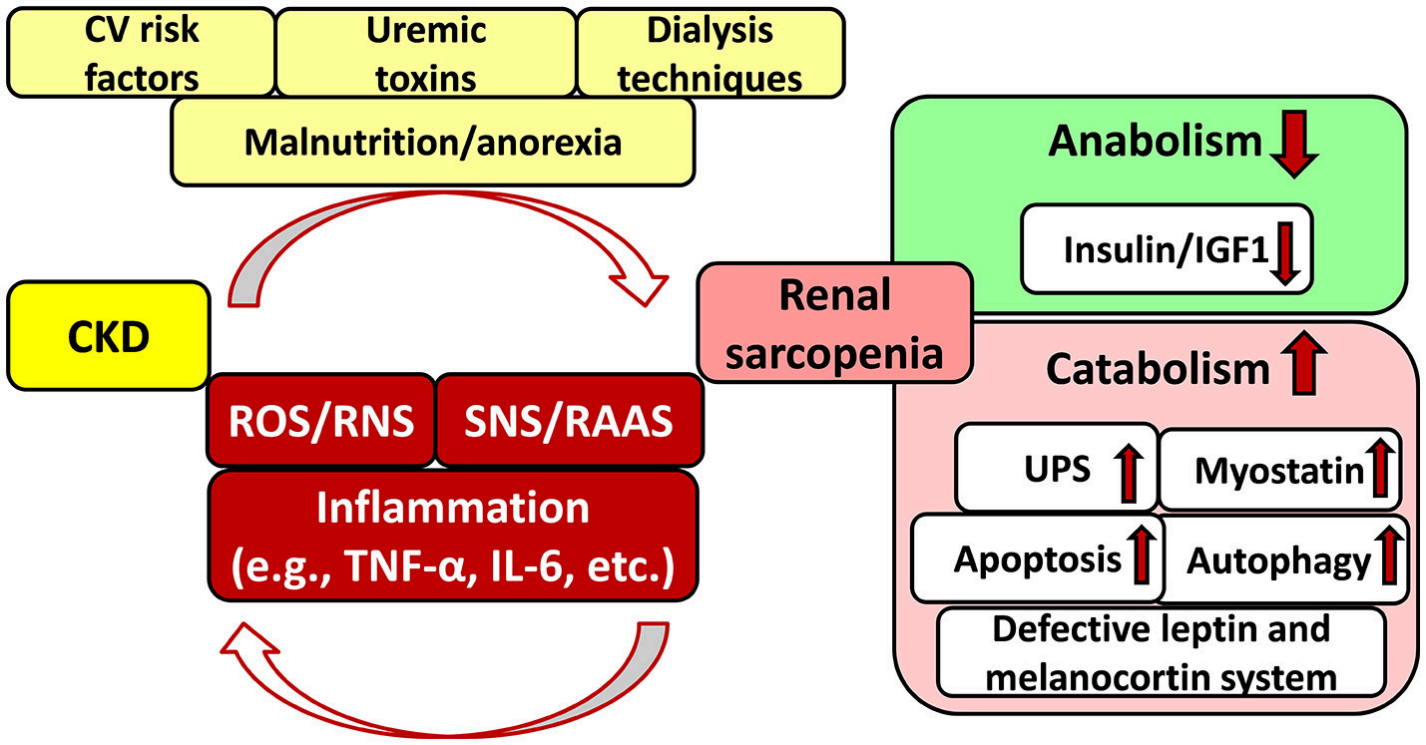
Interplay between chronic kidney disease and sarcopenia. CKD, chronic kidney disease; CV, cardiovascular; IGF1, insulin-like growth factor 1; IL-6, interleukin-6; RAAS, renin–angiotensin–aldosterone system; RNS, reactive nitrogen species; ROS, reactive oxygen species; SNS, sympathetic nervous system; TNF-α, tumor necrosis factor-alpha; UPS, ubiquitin-proteasome system.

CKD is also a catabolic state leading to renal sarcopenia which is characterized by the loss of skeletal muscle strength and physical function (Stenvinkel et al., 2016; Moorthi and Avin, 2017) (Figure 2). Catabolic pathways include e.g., activation of the ubiquitin-proteasome system, caspase-3-mediated apoptosis, autophagy, imbalance between the anabolic insulin/IGF1 and catabolic myostatin signaling pathways, IL-6 and TNF-α-mediated inflammatory pathways, defective leptin signaling and hypothalamic melanocortin systems, over-activated SNS and RAAS, etc. (Mitch and Du, 2004; see for detailed reviews: Mendler et al., 2007; Wang and Mitch, 2014; Yoshida and Delafontaine, 2015; Powers et al., 2016; Kocsis et al., 2017) (Figure 2). These pathways might activate each other and lead to CKD-associated complications including, e.g., hypertension, insulin resistance, metabolic changes, chronic inflammatory state, malnutrition etc (Mitch and Du, 2004; Wang and Mitch, 2014; Yoshida and Delafontaine, 2015; Powers et al., 2016) (Figure 2). Sarcopenia is common and strongly associated with morbidity and mortality in CKD patients especially in those with stage G3, G4, and G5 including ESRD (GFR < 45 mL/min/1.73 m^2^) (Carrero et al., 2013; Wang and Mitch, 2014; Obi et al., 2015). In renal sarcopenia, the loss of skeletal muscle strength leads to easy fatigability (Sakkas et al., 2003; Kaltsatou et al., 2015). It can be caused by the loss and/or atrophy of the muscle fibers (Sakkas et al., 2003; Kaltsatou et al., 2015). In ESRD patients, atrophy and loss of type IIα and IIx fibers, decreased capillarization of fibers, the functional deterioration in the existing muscle mass, and reduced mitochondrial respiratory capacity have been described (Sakkas et al., 2003; Kaltsatou et al., 2015; Roshanravan et al., 2017).

### Major factors in the cardio-reno-muscular crosstalk in CKD

Although CKD and CVD patients share many traditional cardiovascular risk factors including e.g., hypertension, obesity, dyslipidemia, diabetes mellitus, age, etc. these fail to give full explanation for the disproportionately increased cardiovascular mortality risk of CKD patients as compared to the general population (Baigent et al., 2000; Ronco et al., 2008; Kumar et al., 2014; Ortiz et al., 2014) (Figure 1). Both experimental and clinical studies proved that non-traditional cardiovascular risk factors specific to CKD provoked the development of LVH, independently of pressure- and volume-overload induced by the over-activated SNS and RAAS due to renal hypoperfusion (Raev, 1994; Nielsen et al., 1998; McMahon et al., 2000; Wolf et al., 2000; Alhaj et al., 2013) (Figure 1). These non-traditional and CKD-specific risk factors include e.g., uremic toxins, hyperkalemia, and hypocalcemia, metabolic acidosis, abnormal apolipoprotein levels, enhanced coagulability, anemia, endothelial dysfunction, increased plasma homocysteine concentration, elevated systemic and tissue levels of inflammatory factors, protein-energy wasting, oxidative/nitrative stress, etc. (Parfrey et al., 1995; Fort, 2005; Schiffrin et al., 2007; Mitsnefes et al., 2018) (Figure 1). All of these factors might adversely affect muscle mass, performance and exercise tolerance leading to renal sarcopenia (Negrao and Middlekauff, 2008; Bacurau et al., 2016) (Figure 2). Moreover, renal sarcopenia might lead to physical inactivity which promotes the development of cardiovascular complications including acute myocardial infarction and chronic heart failure in CKD patients (Wilund et al., 2010; Moorthi and Avin, 2017). Heart failure developed in T4CRS could also result in chronic muscle hypoperfusion and/or metabolic disturbances (Negrao and Middlekauff, 2008; Bacurau et al., 2016). Although a myriad of factors might contribute to the crosstalk between T4CRS and renal sarcopenia, the precise molecular mechanisms that induce and maintain these pathological conditions are not fully understood yet.

Accumulating evidence suggests that elevated oxidative/nitrative stress could be a key factor in the development of complex biochemical, structural and functional changes associated with T4CRS and renal sarcopenia. Understanding the complex interactions between oxidative/nitrative stress and other pathological pathways (e.g., inflammation, cardiomyocyte hypertrophy, fibrosis, cell death, etc.) is essential to develop novel targeted therapies for T4CRS and renal sarcopenia patients. In this review, we focus on the role of cardiac and skeletal muscle oxidative/nitrative stress in the development of T4CRS and renal sarcopenia. We also review the effects of some clinically used and novel pharmaceuticals, nutraceuticals, and physical exercise on cardiac and skeletal muscle oxidative/nitrative stress in T4CRS and renal sarcopenia.

## Oxidative/nitrative stress and cellular damage

**Figure 3:**
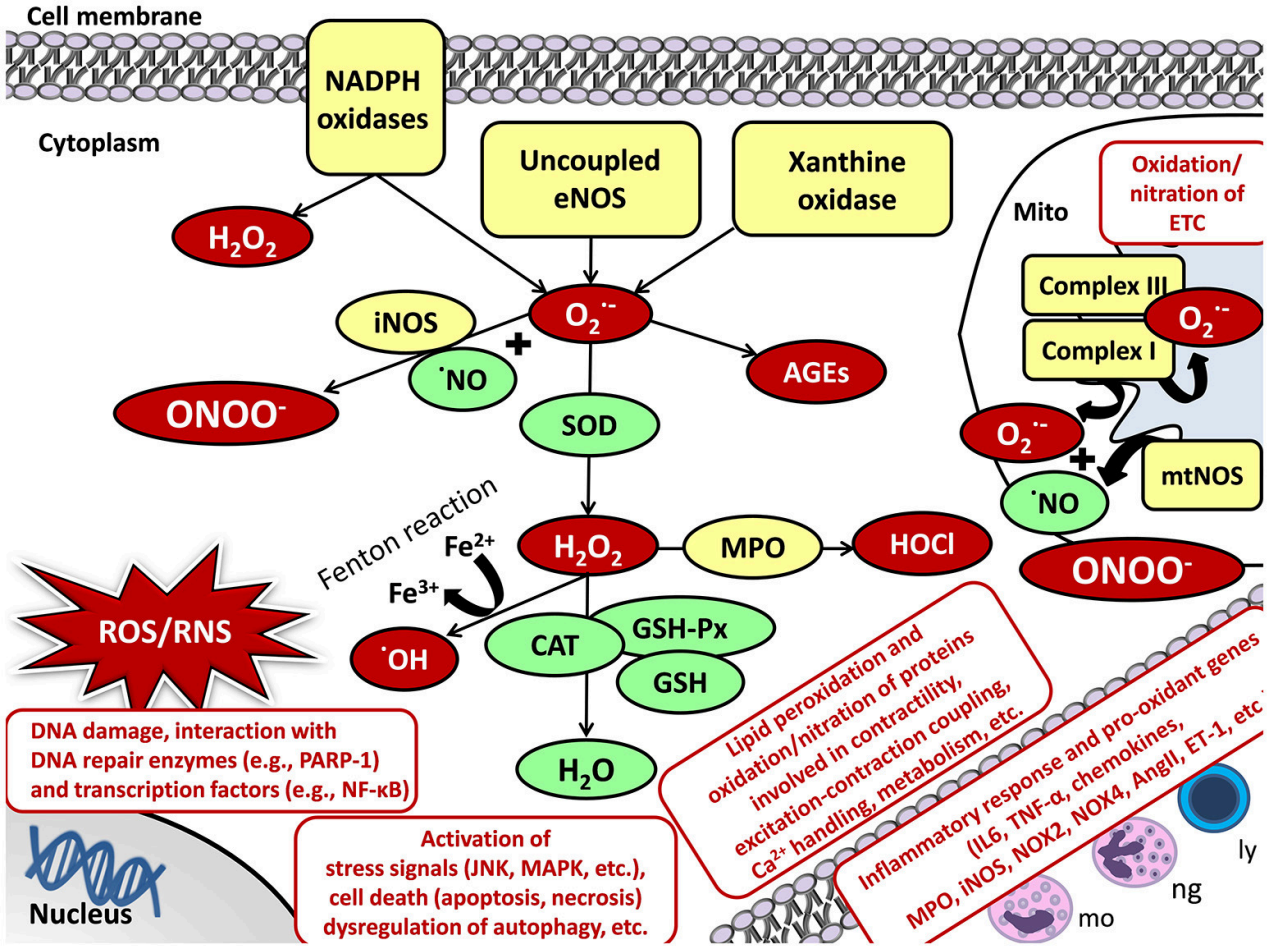
Oxidative/nitrative stress and cellular damage in the heart and skeletal muscle. AngII, angiotensin II; CAT, catalase; DNA, deoxyribonucleic acid; ETC, electron transport chain; ET-1, endothelin-1; Fe^2+/3+^, iron ions; GSH, glutathione; GSH-Px, glutathione peroxidase; H_2_O, water; H_2_O_2_, hydrogen peroxide; HOCl, hypochlorous acid; iNOS, inducible nitric oxide synthase; JNK, c-Jun N-terminal kinase; ly, lymphocytes; MAPK, mitogen-activated protein kinase; mo, monocytes; MPO, myeloperoxidase; mtNOS, mitochondrial nitric oxide synthase; NADPH oxidase, nicotinamide adenine dinucleotide phosphate; NF-κB, nuclear factor kappa-light-chain-enhancer of activated B cells; ng, neutrophil granulocytes; NO, nitric oxide; NOX4, NADPH oxidase 4; ${\text{O}}_{2}^{\cdot -}$, superoxide; OH, hydroxyl radical; PARP-1, poly-ADP-ribose-polymerase 1; RNS, reactive nitrogen species; ROS, reactive oxygen species; TNF-α, tumor necrosis factor-alpha.

Oxidative/nitrative stress is characterized by increased levels of ROS and RNS including, e.g., superoxide (O2^·−^), hydrogen peroxide (H_2_O_2_), hydroxyl radical (^·^OH), and peroxynitrite (ONOO^−^) (Sies, 1997; Sárközy et al., 2016; Radi, 2018). The reason for the development of increased oxidative/nitrative stress could be the excessive production and/or the unsatisfactory removal by the antioxidant defense mechanisms of the highly reactive ROS/RNS molecules (Sies, 1997; Sárközy et al., 2016; Radi, 2018) (Figure 3).

### Cellular sources of ROS/RNS

The main enzymatic sources for ROS/RNS production are the mitochondrial respiratory chain, NADPH oxidases, uncoupled NOS, xanthine oxidase (XO), heme oxygenase (HO), cyclooxygenase, lipoxygenase, cytochrome P450 system, myeloperoxidase, etc. (Di Meo et al., 2016; Radi, 2018). Under physiological conditions, a low amount of ROS/RNS is constantly produced by the mitochondria (Bartz et al., 2015). Mitochondrial ROS production is mainly in the form of O2^·−^ via a single electron transport to molecular oxygen in the respiratory chain at complexes I and III (Bartz et al., 2015) (Figure 3). Mitochondrial RNS production is mainly in the form of ONOO^−^ when O2^·−^ reacts with NO produced by mitochondrial nitric oxide synthase (mtNOS) or inducible nitric oxide synthase (iNOS) isoforms (see for detailed review: Shvedova et al., 2018) (Figure 3). Under normal conditions, mitochondrial O2^·−^ is degraded spontaneously or by mitochondrial superoxide dismutase (SOD2 isoform) to H_2_O_2_. It could either turn to ^·^OH and water via the Fenton reaction or move from the mitochondrion to the cytoplasm (Bartz et al., 2015; Di Meo et al., 2016). Here, it could be catalyzed to water and oxygen by catalase (CAT), glutathione peroxidases (GSH-Px), glutathione (GSH), peroxiredoxins, etc. (Bartz et al., 2015; Di Meo et al., 2016) (Figure 3).

Evidence suggests an interplay between mitochondria and other cellular ROS/RNS sources (Di Meo et al., 2016). Thus, the activation of one can result in the activation of other ROS/RNS sources (Di Meo et al., 2016). In the heart and skeletal muscle, the transmembrane NADPH oxidases (NOXs) also play an important role in the cytoplasmic and mitochondrial ROS production (Rubattu et al., 2013). NADPH serves as an electron donor to reduce oxygen and produce O2^·−^ or H_2_O_2_ (Sirker et al., 2011; Schramm et al., 2012; Hafstad et al., 2013) (Figure 3). Out of the seven NADPH oxidase family members, NOX2 and NOX4 are the main isoforms in cardiomyocytes and skeletal muscle (Sirker et al., 2011; Schramm et al., 2012; Hafstad et al., 2013; Ferreira and Laitano, 2016). NOX2 is present in the plasma membrane and phagosome membranes (Altenhöfer et al., 2012). NOX4 is localized in the plasma membrane, mitochondrial, and endoplasmic reticulum membranes and nucleus (Altenhöfer et al., 2012). XO is another significant cytoplasmic source of ROS which produces O2^·−^ by catalyzing the reaction of hypoxanthine to xanthine and then uric acid in the last phase of purine metabolism both in the cardiomyocytes and skeletal muscle cells (Wilson et al., 2017). In the cytoplasm, O2^·−^ and H_2_O_2_ could be either eliminated by the above mentioned enzymatic (e.g., SOD, CAT, etc.) and non-enzymatic (e.g., glutathione, vitamins A, C, and E, etc.) antioxidant defense mechanisms, or react with NO to form the highly reactive ONOO^−^ (see for detailed review: Pacher and Szabo, 2008) (Figure 3). NO is produced by different isoforms of nitric oxide synthase (NOS). Both in the heart and skeletal muscle, type I or neuronal nitric oxide synthase (nNOS) and type III or endothelial nitric oxide synthase (eNOS) are constitutively expressed under normal circumstances generating low levels of NO (Modlinger et al., 2004; Carnicer et al., 2013; Eghbalzadeh et al., 2014). Under pathophysiological conditions, uncoupling of eNOS from its cofactor tetrahydrobiopterin (TB4) leads to oxidative stress since uncoupled NOS produces O2^·−^ instead of NO and decrease the bioavailability of NO (Pacher and Szabo, 2008). Type II or inducible nitric oxide synthase (iNOS) is robustly up-regulated under pathophysiological circumstances (e.g., inflammation) markedly increasing NO levels which further reacts with O2^·−^ producing ONOO^−^ (Modlinger et al., 2004; Carnicer et al., 2013; Eghbalzadeh et al., 2014) (Figure 3).

### Cellular damage caused by ROS/RNS

Under pathophysiological conditions, high levels of ROS/RNS cause lipid peroxidation, protein oxidation/nitration, inactivation of enzymes, DNA damage, interacts with both DNA repair enzymes (e.g., poly [ADP-ribose] polymerase 1 [PARP-1]) and transcription factors (e.g., NF-κB), lead to the activation of inflammatory response (e.g., IL-1, IL-6, TNF-α, TGF-β, etc.), stress signals (e.g., c-Jun N-terminal kinase [JNK], p38-MAPK) or cell death via different mechanisms (e.g., apoptosis, necrosis, etc.), dysregulation of autophagy, etc. (see for reviews: Graziewicz et al., 2002; Maack and Böhm, 2011; Osterholt et al., 2013; Rubattu et al., 2013; Varga et al., 2015) (Figure 3). In the heart and skeletal muscle, the aforementioned mechanisms and the oxidation/nitration of proteins involved in contractility, excitation-contraction coupling, Ca^2+^ handling, metabolism, elements of the mitochondrial electron transport chain and Krebs cycle, extracellular matrix, etc. might result in deleterious events (Graziewicz et al., 2002; Maack and Böhm, 2011; Osterholt et al., 2013; Rubattu et al., 2013; Varga et al., 2015) (Figure 3). Evidence suggests that chronic activation of RAAS and SNS in heart failure also stimulates inflammation and oxidative/nitrative stress which factors further aggravate each other in both the heart and the kidney (Nakamura et al., 2002; Heymes et al., 2003; Bleeke et al., 2004; Wassmann et al., 2004). Angiotensin II has reported to activate both cardiac and renal NADPH oxidase and subsequently the overproduction of ROS/RNS, which in turn trigger the production of pro-inflammatory mediators including e.g., IL-1, IL-6, TNF, TGF-β, etc. contributing to cardiac and renal fibrosis (Lodha et al., 2002; Schultz et al., 2002; Ijsselmuiden et al., 2008; Zhang et al., 2012).

## Pathways of systemic oxidative/nitrative stress in CKD

It is established that the interplay of multiple factors (e.g., impairment of renal function, comorbidities, over-activated RAAS, elevated oxidative/nitrative stress, inflammation, metabolic changes, etc.) could lead to the development and progression of CKD (see for reviews: Rubattu et al., 2013; Duni et al., 2017). These factors could activate and potentiate each other leading to a vicious cycle. Among these factors, increased oxidative/nitrative stress plays a key role in the complex pathomechanism of CKD. In this section, we summarize the major factors of CKD-induced systemic oxidative/nitrative stress and some clinically relevant mechanisms which interact with oxidative/nitrative stress in CKD.

### Over-production of ROS/RNS vs. ineffective antioxidant defense mechanisms in CKD

Elevated systemic oxidative/nitrative stress is believed to be mainly the consequence of higher ROS/RNS production in CKD (Bossola and Tazza, 2015; Duni et al., 2017). Reduced clearance of pro-oxidant substances due to renal dysfunction and impaired antioxidant defense mechanisms supposed to play a complementary role (Duni et al., 2017). The over-production of ROS/RNS is aggravated by patient-related (e.g., age, uremic toxins, chronic systemic inflammation, pro-oxidant comorbidities including obesity, hypertension, diabetes mellitus, etc.), and hemodialysis-related factors (e.g., membrane bio-incompatibility, endotoxins, etc.) especially in ESRD patients on long-term dialysis (Bossola and Tazza, 2015) (Figure 2). The impairment of antioxidant defense mechanisms (e.g., decreased levels of vitamin C and E, deficiency in the glutathione scavenging system, etc.) become more pronounced only in ESRD (Bossola and Tazza, 2015). Nuclear factor-erythroid-2-related factor 2 (Nrf2) is a transcription factor which coordinates the induction of several genes including antioxidant and detoxifying enzymes and associated proteins (Kim and Vaziri, 2010; Ruiz et al., 2013). Experimental studies have demonstrated that CKD animals showed a significant and time-dependent decrease in nuclear Nrf2 amount, despite the oxidative stress and inflammation, which should have led to Nrf2 activation and overexpression of its target genes (Kim and Vaziri, 2010; Ruiz et al., 2013).

### Increased renal ROS/RNS production as a major cause of systemic oxidative/nitrative stress in CKD

Systemic oxidative/nitrative stress is progressively increased in CKD and is most prominently elevated in ESRD patients (Dounousi et al., 2006; Small et al., 2012; Bossola and Tazza, 2015). In CKD patients, ROS levels in the blood plasma strongly correlate with renal ROS production and NOX4 activity which is thought to be the major source of ROS in the kidneys (Vaziri et al., 2007; Duni et al., 2017). NOX4 is also the predominant form of NADPH oxidases in the mitochondrial membrane (Vaziri et al., 2007; Duni et al., 2017). The main pathophysiological mechanism underlying the renal overproduction of ROS is supposed to be the upregulation of the intrarenal RAAS (Sedeek et al., 2013; Duni et al., 2017). It has been demonstrated that angiotensin II-mediated activation of the AT1 receptor could increase the renal and vascular O2^·−^ production via NOX isoforms (Vaziri et al., 2007; Kim et al., 2011; Duni et al., 2017). It has also been proposed that increased oxidative/nitrative stress itself results in accelerated progression of the kidney disease via cytotoxic mechanisms perpetuating a vicious cycle (Dounousi et al., 2006; Bossola and Tazza, 2015; Duni et al., 2017).

### The interplay of increased systemic oxidative/nitrative stress with chronic inflammation in CKD

Chronic inflammation is thought to be the most common result of the elevated oxidative/nitrative stress in CKD as a consequence of the activation of nuclear factor-kB (NF-kB) and other transcription factors regulating the gene expression of pro-inflammatory cytokines and chemokines (Jin Jung et al., 2013; Tucker et al., 2015; Wu et al., 2016; Duni et al., 2017). Moreover, both oxidative/nitrative stress and inflammation can amplify each other (Raizada et al., 2007; Jin Jung et al., 2013; Tucker et al., 2015; Wu et al., 2016; Duni et al., 2017). On the one hand, elevated oxidative/nitrative stress stimulates recruitment and activation of leukocytes, the formation of pro-inflammatory oxidized lipids, advanced protein oxidation products, and advanced glycation end products (AGEs) (Duni et al., 2017). On the other hand, activated leukocytes, macrophages, and other cells generate ROS/RNS which further enhances oxidative/nitrative stress (Fortuno et al., 2005; Duni et al., 2017). Indeed, a study reported that ROS generated by NADPH oxidase was enormously elevated in circulating lymphocytes and monocytes even in the early stages of CKD (Fortuno et al., 2005; Duni et al., 2017). CKD is characterized by chronic inflammation with higher plasma concentrations of C-reactive protein (CRP), interleukin-6 (IL-6), tumor necrosis factor a (TNF-α) and fibrinogen, and lower levels of albumin (Kumar et al., 2014). ROS such as O2^·−^ and H_2_O_2_ activate NF-kB, which is inactive in the cytoplasm of normal cells, including renal cells, endothelial cells, smooth muscle cells, cardiac myocytes, etc. (Raizada et al., 2007). Activated NF-kB upregulates endothelin production and genes encoding proteins involved in apoptosis, cell growth (e.g., fibroblast growth factors [FGFs], transforming growth factor-beta [TGF-β], platelet-derived growth factors [PDGF], vascular endothelial growth factor [VEGF], etc.), inflammation (e.g., vascular cell adhesion protein 1 [VCAM1], intercellular adhesion molecule 1 [ICAM1], monocyte chemoattractant protein 1 [MCP1], etc.), coagulation (e.g., plasminogen activator 1, tissue factor, etc.,) and extracellular matrix deposition (e.g., collagen-1, fibronectin, etc.) leading to fibrosis and end-organ damage in the kidney, heart, vasculature, etc. (Raizada et al., 2007). Moreover, NF-kB could upregulate the iNOS gene resulting in excessive NO production which easily forms ONOO^−^ reacting with O2^·−^ (Raizada et al., 2007).

### Systemic oxidative/nitrative stress and atherosclerosis in CKD

As the renal function progressively declines, the level of the anti-atherogenic high-density lipoprotein (HDL) also declines and the produced form is dysfunctional due to the reduced synthesis of its component apolipoprotein-A1 (apo-A1) in the liver (Schiffrin et al., 2007). Moreover, higher levels of the atherogenic low-density lipoprotein (LDL) cholesterol and lipoprotein(a) [Lp(a)] is found with declining of the kidney function (Baigent et al., 2000; Kumar et al., 2014). Accumulation of LDL cholesterol activates the RAAS and also induces the overexpression of the angiotensin type 1 receptor (AT1) further aggravating oxidative/nitrative stress and inflammation resulting in endothelial dysfunction and accelerated atherosclerosis (Kaysen and Eiserich, 2004; Methe and Weis, 2007; Schiffrin et al., 2007; Kumar et al., 2014). Angiotensin II causes hypertension not only via vasoconstriction and atherosclerosis but also activates vascular NADPH oxidase which produces O2^·−^ (Methe and Weis, 2007; Kumar et al., 2014). It inactivates NO via the formation of ONOO^−^ leading to smooth muscle hypertrophy and proliferation, hypertension, atherosclerosis and heart failure (Methe and Weis, 2007; Kumar et al., 2014). Another major player in the acceleration of vascular injury and atherosclerosis is the elevated level of myeloperoxidase (MPO) which is present in leukocytes, monocytes and tissue macrophages, and promotes the peroxidation of LDL cholesterol (Schiffrin et al., 2007; Kumar et al., 2014). The chronic inflammatory state also triggers vascular injury and premature atherosclerosis which is more characteristic in younger CKD patients (Kumar et al., 2014). In turn, dyslipidemia is also an important factor in the development of the chronic inflammatory state and atherosclerosis (Kaysen and Eiserich, 2004).

## Pathways of cardiac oxidative/nitrative stress in T4CRS

Abundant data have linked the cardiac overproduction of ROS/RNS to the development of LVH and heart failure in preclinical and clinical studies (see for reviews: Ungvári et al., 2005; Maack and Böhm, 2011; Osterholt et al., 2013; Varga et al., 2015). However, most of the experimental studies regarding the role of cardiac ROS/RNS in the development of LVH and heart failure used hypertension-induced or diabetic models. In T4CRS, CKD-specific factors and mechanisms might also contribute to the development of LVH and heart failure. There are only a limited number of studies available in the literature which investigates the role of oxidative/nitrative stress in the development of LVH and heart failure in CKD. Therefore, in this section, we collected studies which describe both renal and cardiac function and/or morphology and investigate the role of ROS/RNS in the development of uremic cardiomyopathy.

### Cardiac oxidative/nitrative stress in T4CRS in preclinical studies

Based on experimental studies investigating the role of CKD-induced cardiac oxidative/nitrative stress, the over-activation of NADPH-oxidase (Goux et al., 2011; Suematsu et al., 2018), especially its NOX4 (Bai et al., 2009; Fukunaga et al., 2012; Kuczmarski et al., 2014; Han et al., 2015; Liu et al., 2015) and NOX2 isoforms (Yin et al., 2016), uncoupled eNOS and iNOS (Chang et al., 2015; Oosterhuis et al., 2017) are the main sources of increased cardiac ROS/RNS production in T4CRS. Mitochondrial dysfunction is also supposed to play a major role in the development of heart failure in T4CRS (Correa et al., 2013; Hernandez-Resendiz et al., 2015; Taylor et al., 2015). Chang et al. found that mild CKD induced by unilateral nephrectomy impaired cardiac relaxation in Sprague-Dawley rats (Chang et al., 2015). They also showed that impaired cardiac relaxation was associated with increased NO-dependent nitrosylation of sarcoendoplasmic reticulum calcium ATPase 2a (SERCA2a) due to increased iNOS expression and uncoupled eNOS (Chang et al., 2015). The reduced cardiac bioavailability of NO is also described in T4CRS (Chen et al., 2014). Some studies reported impaired antioxidant defense mechanisms in the heart in T4CRS. According to these studies, reduced SOD (Sener et al., 2004a,b; Chen et al., 2014; Kuczmarski et al., 2014; Suematsu et al., 2018), and CAT (Sener et al., 2004a; Bock and Gottlieb, 2010) activities; (García-Trejo et al., 2016) and reduced cellular GSH (Sener et al., 2004b, 2007) and GST (Correa et al., 2013) levels seems to be characteristic features of uremic cardiomyopathy.

### Oxidative/nitrative stress in T4CRS in clinical studies

There are only a limited number of clinical studies investigating the direct link between oxidative/nitrative stress and the development of LVH and heart failure in T4CRS. In a clinical cohort study, plasma GSH levels correlated with the degree of cardiac dysfunction checked by speckle tracking echocardiography in children with ESRD (Al-Biltagi et al., 2016). In another study, a cross-sectional analysis recruiting 78 adults with stage 3-4 CKD (eGFR: 25–60 mL/min) revealed that elevated plasma F2-isoprostane level as a marker of oxidative stress was associated with reduced heart rate variability (Fadaee et al., 2017). Reduced heart rate variability is a marker of cardiac autonomic dysfunction and a risk factor for fatal arrhythmias and sudden cardiac death (Fadaee et al., 2017). CKD patients often have lipoprotein abnormalities including, e.g., increased remnant particles, triglycerides, and oxidized LDL as a byproduct of oxidative stress, and both deficiency and dysfunction of HDL which are well-known cardiovascular risk factors (Kennedy et al., 2013). Paraoxonase (PON-1) is an HDL-associated glycoprotein and is believed to be responsible for several systemic antioxidant activities of HDL, including, e.g., protective effects against oxidation of lipoproteins and phospholipids (Kennedy et al., 2013). Serum PON-1 function is characterized by its arylesterase activity. In a prospective cohort study recruiting 630 CKD patients has been shown that decreased serum arylesterase activity predicted higher risk for long-term cardiovascular events (e.g., heart attack, stroke, or death) (Kennedy et al., 2013).

## Modulation of oxidative/nitrative stress in T4CRS

Modulation of oxidative/nitrative stress in T4CRS can be achieved by different approaches. Theoretically, the prevention or treatment of CKD could be the most effective way to eliminate its oxidative/nitrative stress triggering effect and cardiovascular complications. However, CKD is a chronic, progressive disease and the maintenance or prevention of its progression is a major clinical challenge. To the best of our knowledge, there are no pharmaceutical approaches in the clinical practice to specifically target cardiac oxidative/nitrative stress in T4CRS. A therapeutic option is that several well-known pharmaceuticals used for the treatment of the cardiovascular complications of CKD have a complex mechanism of action which might include antioxidant properties. Another therapeutic option could be the administration of antioxidant molecules and natural products to decrease cardiac oxidative/nitrative stress. There is a growing interest in nutraceuticals instead of chemically synthesized compounds to treat or prevent certain diseases by modulating oxidative/nitrative stress. Nutraceuticals include a wide range of products including, e.g., dietary supplements, natural herbal products, isolated nutrients, etc. (Csonka et al., 2016). Nutraceuticals could have anti-oxidant properties due to their unique composition of different antioxidant compounds (Csonka et al., 2016). The third therapeutic option is the induction of endogenous enzymatic antioxidant mechanisms or blockage of the pro-oxidant enzymes, which might be appropriate ways to decrease the systemic and cardiac oxidative/nitrative stress in T4CRS. These therapeutic options could also be combined. In this section, we focus on various pharmaceuticals, nutraceuticals, some novel therapeutic modalities, and even physical exercise as possible modulators of CKD-induced oxidative/nitrative stress in T4CRS.

### Pharmaceuticals modulating oxidative/nitrative stress in T4CRS in preclinical studies

#### Angiotensin II type 1A receptor blockers in single or combination therapy

It has been shown that 12-week treatment with the AT1 receptor blocker valsartan (sc. 1 mg/kg/day administered by osmotic minipumps) or AT1 KO genotype could improve cardiac remodeling, both systolic and diastolic dysfunction and oxidative DNA damage in partially nephrectomized mice (Li et al., 2007) (Table 1). A novel therapeutic approach in the therapy of chronic heart failure is the increase of the levels of vasoactive peptides including natriuretic peptides (A-type, B-type and C-type natriuretic peptides) which can ameliorate cardiac hypertrophy and remodeling (Li et al., 2007; Suematsu et al., 2018). The expression of these vasoactive peptides can be increased via the blockage of neprilysin which is a key enzyme in the breakdown of these peptides (Suematsu et al., 2018). Combined therapy with the neprilysin inhibitor prodrug sacubitril and AT1 blocker valsartan (60 mg/kg) for 8 weeks could attenuate cardiac hypertrophy and fibrosis in rats with 5/6 nephrectomy (Suematsu et al., 2018) (Table 1). In this study, the combination therapy could decrease the left ventricular levels of NOX4, MPO, and the NADPH-oxidase subunit gp91^phox^ in the partially nephrectomized rats (Suematsu et al., 2018) (Table 1). Moreover, it could increase the levels of eNOS, Nrf2, Cu-SOD, Zn-SOD, HO-1, CAT, GSH-Px, and ATP-synthase in rats with CKD (Suematsu et al., 2018) (Table 1). It has been reported that cardiac function and fibrosis could be improved by the AT1 blocker losartan or combination treatment (NF-inhibitor PDTC + NO-donor molsidomine + SOD mimetic tempol) or losartan + combination treatment + metoprolol (Table 1). However, renal fibrosis was most effectively decreased by the losartan + combination treatment + metoprolol in this study (Oosterhuis et al., 2017).

**Table 1:** Pharmaceuticals modulating oxidative/nitrative stress in T4CRS in preclinical studies.

	**Experimental model**	**Age/body weight**	**FUP time**	**Characteristics of HF**	**Molecular markers of HF**	**Markers of cardiac oxidative/nitrative stress**	**Protective agent**	**References**
1	AT1 KO and WT mice (C57BL/6 background)	10 weeks	12 weeks	↑ LVSP, ↓ EF and ±dP/dt, ↓ capillary density myocardial fibrosis	↑ MMP-2 and 9, ↓ TIMP-1, ↑ TGF-β, ↑ PI3K/Akt pathway,	↑ DNA and cell membrane oxidative damage	AT1 receptor blockade (valsartan)	Li et al., 2007
2	5/6 STNX male Sprague-Dawley rats	N/A	8 weeks	↑ HW/BW ↑ cross sectional area of cardiomycytes, ↑ SBP, cardiac and aortic fibrosis,	↓ mitochondrial mass, ↓ ATP-synthase ↑β-MHC, tropomyosin, ↑ collagen, TGFβ, FGF-23	↑cardiac protein carbonyl content; ↑ MCP-1, MPO, COX-2, ↑ NOX4, gp91 phox, ↓ Cu/Zn SOD, CAT, GSH-Px, HO-1, eNOS	LCZ696 (Sacubitril/ valasrtan)	Suematsu et al., 2018
3	Male Lewis rats with chronic renocardiac syndrome (5/6 STNX, fed with 6% NaCl supplemented chow, left coronary ligation)	180-200 g	16 weeks	↓ EF, ↓ heart rate; ↓ cardiac output; ↓ stroke volume	↑ cardiac BNP mRNA, ↑ cardac fibrosis area	thiobarbituric acid reactive substances excretion in urine	losartan; PDTC + molsidomine + tempol; PDTC + molsidomine + tempol + metoprolol	Oosterhuis et al., 2017
4	5/6 STNX male Sprague-Dawley rats	200–250 g	6 weeks	↑ HW/BW ↑ SBP ↑ cross sectional area of cardiomyocytes interstitial fibrosis and ECM deposition	↑ mRNA levels of TGF-β, collagen I and III, TNF-α, IL-6, MCP-1 ↑ phospho-ERK1/2, p38 MAPK	↑ expressions of gp91phox, p47phox, p67phox	Renalase	Yin et al., 2016
5	5/6 STNX male Lewis rat fed with NaCl enriched chow	180–200 g	8 weeks	↑ LV mass, ↑ EDV and ESV, ↓ EF and cardiac output, ↑ SBP, DBP,	N/A	↑ nNOS	Molsidomine	Bongartz et al., 2010
6	5/6 STNX male Wistar rats	200–250 g	4 weeks	fibrosis; degenerated cardiac muscle with vascular congestion	↑ collagen content	↓ plasma antioxidant capacity, ↓ cellular GSH level, ↑ increased lipid peroxidation,	Montelukast	Sener et al., 2007
7	Doxorubicin+right NTX male Lewis rat	8-week-old	20 weeks	LVH, myocardial and perivascular fibrosis	↑ collagen content	↑ number of 8-OHdG- and acrolein-posive cells	AST-120	Fujii et al., 2009
8	5/6 STNX male Wistar rats	200–250 g	4 weeks	N/A	N/A	↑ lipid peroxidation and carbonyl concentration (aorta, heart, plasma) ↓ GSH (aorta, heart, plasma), ↓ plasma SOD, GSH-Px and CAT activity	Melatonin	Sener et al., 2004a

*5/6 STNX, 5/6 subtotal nephrectomy; 8-oxodG, 8-oxo-7,8-dihydro-2'-deoxyguanosine; AT1 KO, angiotensin II receptor type 1 knock out; ATP, adenosine triphosphate; BNP, B-type natriuretic peptide; β-MHC, beta myosin heavy chain; BW, body weight; CAT, catalase; COX-2, cyclooxygenase-2; Cu, copper; DBP, diastolic blood pressure; DNA, deoxyribonucleic acid; ECM, extracellular matrix; EDV, end diastolic volume; EF, ejection fraction; eNOS, endothelial nitric oxide synthase; ERK-1/2, extracellular signal-regulated protein kinases 1 and 2; ESV, end systolic volume; FGF-23, fibroblast growth factor-23; FUP, follow-up; gp91, NOX subunit; GSH, glutathione; GSH-Px, glutathione peroxidase; GST, glutathione S-transferase; IVS, interventricular septum; H_2_O_2_, hydrogen peroxide; HO-1, heme oxygenase-1; HW, heart weight; IL-6, interleukin-6; LV, left ventricular; LVDP, left ventricular diastolic pressure; LVESD, left ventricular end-systolic diameter; LVSP, left ventricular systolic pressure; MCP-1, monocyte chemoattractant protein-1; MMP, matrix metalloprotease; MPO, myeloperoxidase; NaCl, Natrium chloride; NOX4, NADPH oxidase 4; p38 MAPK, p38 mitogen-activated protein kinase; PI3K/Akt, phosphoinositide 3-kinase/Akt pathway; SBP, systolic blood pressure; SOD, superoxide dismutase; TGF-β:transforming growth factor-beta; TIMP, tissue inhibitor of matrix metalloprotease; Zn, zinc*.

#### Renalase

Circulating catecholamines could be degraded by renalase which is a soluble mono-amino oxidase secreted by the kidneys (Yin et al., 2016). It has been demonstrated that renalase supplementation by adenoviral transduction attenuated hypertension, LVH, fibrosis and the cardiac expression of NADPH-oxidase components gp91^phox^, p47^phox^, and p67^phox^ in partially nephrectomized rats (Yin et al., 2016) (Table 1).

#### No donor molsidomine

Bongartz et al. found that 5/6 nephrectomy impaired left ventricular systolic function in male inbred Lewis rats fed with NaCl enriched chow; however, molsidomine (NO donor) treatment for 5 weeks improved heart function and creatinine clearance (Bongartz et al., 2010). Molsidomine increased NO metabolite excretion but did not increase 3-nitrotyrosine content in the heart, liver or kidney tissues (Bongartz et al., 2010) (Table 1). In this study, the cardiac expression of constitutive isoforms of NOS (nNOS, eNOS) was not changed; however, iNOS expression was decreased by molsidomine (Bongartz et al., 2010) (Table 1).

#### CysLT1 receptor antagonist montelukast

Four-week treatment with the selective CysLT1 receptor antagonist anti-inflammatory drug montelukast (10 mg/kg i.p.) could reduce cardiac fibrosis, inflammation, MPO and MDA levels and increase cardiac glutathione levels and total antioxidant capacity in male 5/6 nephrectomized rats (Sener et al., 2007) (Table 1).

#### Oral charcoal adsorbent AST-120

Twenty-week treatment with the oral charcoal adsorbent AST-120 which reduces the levels of circulating uremic toxins (e.g., indoxyl sulfate) could improve LVH, fibrosis and systemic oxidative stress in doxorubicin plus right nephrectomy-induced CKD in rats (Fujii et al., 2009) (Table 1).

#### Melatonin

Treatment with the pineal gland hormone melatonin (10 mg/kg i.p.) for 4 weeks was also reported to reduce cardiac MDA level and protein carbonylation and increase cardiac GSH levels in male 5/6 nephrectomized rats due to its antioxidant and free radical scavenging features (Sener et al., 2004a) (Table 1).

## Pharmaceuticals modulating oxidative/nitrative stress in T4CRS in clinical studies

### Statins

The cholesterol-lowering drugs statins could markedly reduce the risk of cardiovascular mortality in the general population. In contrast, statins had less, or no benefit on CVD in hemodialysis patients in large clinical trials (4D with 1255 dialyzed T2DM patients, AURORA with 2766 ESRD patients, and SHARP with 3023 dialyzed and 6247 non-dialyzed CKD patients) (Wanner et al., 2005; Fellström et al., 2009; Baigent et al., 2011). Moreover, Wagner et al. found no correlation between serum oxidized LDL level and major cardiac events in both dialyzed and non-dialyzed CKD patients using the data of the AURORA and LURIC studies (Wagner et al., 2017). These results suggest that the development of cardiovascular disease is atypical and complex in CKD.

## Nutraceuticals modulating oxidative stress in T4CRS in preclinical studies

### Curcumin

Curcumin is a yellow pigment with polyphenol structure in curry spice, turmeric, and to lesser extent ginger (Correa et al., 2013). In partially nephrectomized rats, curcumin used for 7 days as a pretreatment before operations and treatment for 60 days could preserve ejection fraction, reduce cardiac lipid peroxidation and increase CAT and glutathione-S-transferase (GST) levels (Correa et al., 2013) (Table 2).

**Table 2:** Nutraceuticals modulating oxidative/nitrative stress in T4CRS in preclinical studies.

	**Experimental model**	**Age/body weight**	**FUP time**	**Characteristics of HF**	**Molecular markers of HF**	**Markers of cardiac oxidative/nitrative stress**	**Protective agent**	**References**
1	5/6 STNX male Wistar rats	280–300 g	60 days	↑ HW/BW, ↑ IVS, ↓ LVDd and LVSd, ↓ EF, ↑ SBP, ↓ LVDP, hypertrophic remodelling	mitochondrial disintegrity, mitochondrial dysfunction	↑ ROS generation, ↑ lipidperoxidation, ↑ protein oxidation, ↓ CAT activity and GST level	Curcumin	Correa et al., 2013
2	5/6 STNX male Wistar rats	280–300 g	6 weeks	↑ LV/BW, ↑ SBP, ↓ coronary perfusion, pressure, ↓ LV performance	N/A	↑ lipid and protein oxidation, ↓ Nrf2, CAT, SOD, GSH-Px	Allicin	García-Trejo et al., 2016
3	5/6 STNX male Wistar rats	200–250 g	4 weeks	-	-	↑ lipid peoroxidation (heart, aorta and plasma),↓ GSH level, (heart, plasma) ↓ plasma SOD and GSH-Px activities	L-Carnitine	Sener et al., 2004b

*5/6 STNX, 5/6 subtotal nephrectomy; BW, body weight; CAT, catalase; DNA, deoxyribonucleic acid; EF, ejection fraction; eNOS, endothelial nitric oxide synthase; FUP, follow-up; GSH, glutathione; GSH-Px, glutathione peroxidase; GST, glutathione S-transferase; IVS, interventricular septum; HO-1, heme oxygenase-1; HW, heart weight. LV, left ventricular; LVDd, left ventricular end-diastolic diameter; LVDP, left ventricular diastolic pressure; LVSd, left ventricular end-systolic diameter; LVSP, left ventricular systolic pressure; ROS, reactive oxygen species; SBP, systolic blood pressure; SOD, superoxide dismutase*.

### Allicin

Treatment with the garlic extract component allicin (40 mg/kg/day p. os) for 6 weeks attenuated hypertension, LVH, cardiac lipid and protein oxidation and elevated the levels of antioxidant enzymes including SOD, CAT, and GSH-Px in male 5/6 nephrectomized rats (García-Trejo et al., 2016) (Table 2).

### L-carnitine

L-carnitine is a cofactor playing a role in the transport of long-chain fatty acids into the mitochondria, where they fuel the beta-oxidation to produce energy for the cell (Hurot et al., 2002). L-carnitine deficiency is uncommon in the healthy population consuming enough protein. In contrast, hemodialysis patients might develop carnitine deficiency due to the elimination of carnitine during dialysis, gastrointestinal malabsorption, altered transport mechanisms through membranes, decreased renal synthesis, or increased carnitine requirements (Hurot et al., 2002). It has been showed that L-carnitine (500 mg/kg i.p.) administration for 4 weeks could reduce the lipid peroxidation marker MDA and increase the antioxidant glutathione level both in the plasma and heart in partially nephrectomized rats (Sener et al., 2004b) (Table 2).

## Nutraceuticals modulating oxidative/nitrative stress in T4CRS in clinical studies

### L-carnitine

A clinical pilot study enrolling 12 ESRD patients showed that L-carnitine (20 mg/kg i.v.) supplementation for 8 weeks increased the time to fatigue, plasma GSH level, and GSH-Px activity and decreased submaximal heart rate, respiratory quotient, and plasma lactate, MDA and protein carbonyl levels after cycling exercise (Fatouros et al., 2010).

### Vitamin E

The SPACE study enrolling 196 ESRD patients found that 800 IU/day vitamin E as antioxidant supplementation could reduce the CVD endpoints and myocardial infarction in hemodialysis patients with pre-existing cardiovascular disease (Boaz et al., 2000).

### Coenzyme Q10 (CoQ_10_)

CoQ_10_ moves electrons from complexes 1 and 2 to complex 3 in the mitochondria (Small et al., 2012). CoQ_10_ depletion might result in disturbances in the mitochondrial electron transport leading to elevated ROS/RNS production and decreased ATP generation (Small et al., 2012). Plasma CoQ_10_ concentration has been shown to be decreased in hemodialysis patients (Rivara et al., 2017). It has been demonstrated in a dose-escalation study recruiting 20 hemodialysis patients that oral CoQ_10_ supplementation for 8 weeks is also safe and well-tolerable in a dose of 1,800 mg/day and could reduce plasma isofuran concentration which is a marker of systemic oxidative stress (Yeung et al., 2015). Another clinical pilot study enrolling 65 hemodialysis patients showed that CoQ_10_ administration for 4 months at a dose of 1,200 mg/day could reduce the plasma levels of F2-isoprostanes which are systemic oxidative stress markers, however, it has no significant effect on the cardiac biomarkers cTnT and NT-pro-BNP (Rivara et al., 2017).

## Novel pharmacological approaches modulating oxidative/nitrative stress in T4CRS in preclinical studies

### Apocynin

Apocynin is an assembly inhibitor of NOX possibly via the inhibition of the expressions of NOX subunits and translocation from the cytoplasm to the membrane (Liu et al., 2015). The cardioprotective effects of apocynin are related to the decrease of NOX-dependent oxidative stress, but the potential downstream mechanisms are not clear yet (Liu et al., 2015). Liu et al. reported that apocynin treatment (dosed in drinking water, 1.5 mmol/L, for 8 weeks) attenuated the oxidative stress-induced ERK1/2 phosphorylation and cardiac fibrosis and HFrEF in 5/6 nephrectomized rats via inhibiting NADPH oxidase (Liu et al., 2015) (Table 3). Apocynin also inhibited the upregulation of the important profibrotic factor FGF-2 (Liu et al., 2015). Zhang et al. also found that apocynin improved cardiac hypertrophy and fibrosis in CKD rats 8 weeks after 5/6 nephrectomy (Zhang et al., 2015). Han et al. showed that treatment with the protein-bound uremic toxin P-cresyl sulfate (PCS) upregulated the protein expression levels of the NADPH oxidase subunits p22^phox^ and p47^phox^ in H9c2 cardiac myoblasts (Han et al., 2015). Moreover, PCS treatment for 8 weeks (100 mg/kg/day p.os) further augmented cardiac fibrosis and diastolic dysfunction in 5/6 nephrectomized mice (Han et al., 2015). The diastolic dysfunction could be partially restored by apocynin in the PCS-treated CKD mice (Han et al., 2015) (Table 3).

**Table 3:** Novel pharmacological approaches modulating oxidative/nitrative stress in T4CRS in preclinical studies.

	**Experimental model**	**Age/body weight**	**FUP time**	**Characteristics of HF**	**Molecular markers of HF**	**Markers of cardiac oxidative/nitrative stress**	**Protective agent**	**References**
1	5/6 STNX male Sprague-Dawley rats, 1–3-day-old neonatal Sprague-Dawley rats (cell culture)	160 ± 20 g	8 weeks	↑ LVPWd, LVESD, ↓ left ventricular fractional shortening and EF	↑ interstitial fibrosis, FGF-2	MDA, superoxide anion, mitochondrial ROS production, NOX activity	Apocynin	Liu et al., 2015
2	5/6 STNX male Sprague-Dawley rats, H9c2 rat cell line	160-180 g	8 weeks	↑ LVPWd, ↓ EF	↑ interstitial fibrosis, ANP, β-MHC, cardiac fibrosis index, cardiac collagen volume fractions	sEH (soluble epoxide hydrolase), EET (epoxyeicosatrienoic acids)	Apocynin	Zhang et al., 2015
3	5/6 STNX male C57BL/6J mice	5 weeks	8 weeks	↓ FS, EF, E/A ratio, ↑ interstitial and perivascular collagen	↑ collagen, caspase-3 activity (apoptosis)	↑ p22phox and p47phox expressions, ↑ intracellular ROS production	Apocynin	Han et al., 2015
4	5/6 STNX male Sprague-Dawley rats	8 weeks	4 weeks	↑ HW/BW, ↑ SBP, ↑ LA cardiomyocyte diameter, ↑ interatrial conduction time and P-wave duration	↑ collagen I and III, α-SMA	↑ protein expression of NOX4, gp91phox, Nox4, p47phox	Sodium zinc dihydrolipoylhis tidinate (DHLHZn)	Fukunaga et al., 2012

*5/6 STNX, 5/6 subtotal nephrectomy; α-SMA, alpha smooth muscle actin; ANP, A-type natriuretic peptide; β-MHC, beta-myosin heavy chain; BW, body weight; E/A, early wave/atrial wave (diastolic parameter); EF, ejection fraction; FGF-2, fibroblast growth factor-2; FS, fractional shortening; FUP, follow-up; HW, heart weight; LA, left atrial; LVSED, left ventricular end-systolic diameter; LVPWd, left ventricular posterior wall thickness in diastole; MDA, malondialdehyde; SBP, systolic blood pressure; p22phox, NOX subunit; p47phox, NOX subunit; ROS, reactive oxygen species*.

### Sodium zinc dihydrolipoylhistidinate

Sodium zinc dihydrolipoylhistidinate (DHLHZn) is a novel Zn^2+^/dihydrolipoic acid derivate antioxidant drug. Left atrium isolated from 5/6 nephrectomized rats showed greater left atrial diameters and fibrosis, increased left atrial protein expression of NOX4 and subunits gp91^phox^ and p47^phox^. DHLHZn treatment (5 mg/kg/day s.c. by osmotic minipump) successfully attenuated the atrial fibrosis and the overexpression of NOX4, gp91^phox^, p47^phox^ in the left atrium (Fukunaga et al., 2012) (Table 3).

### microRNAs (miRs)

It has been recently recognized that small non-coding RNAs including miRs (approximately 18–25 nucleotides in length) play a myriad of roles in physiological and pathophysiological mechanisms by repression of the translation or destabilization of the mRNA of various target genes (Djebali et al., 2012). Therefore, they might act as fine tuners or on/off switches of the gene expression (Djebali et al., 2012). Certain miRs (e.g., miR-1; miR-25, miR-21, miR-210, the miR-200 family, miR-212, miR-181a, etc.) have been implicated in cellular oxidative/nitrative stress response in CVD in experimental studies (Magenta et al., 2013; Varga et al., 2013; Yildirim et al., 2013; Azzouzi et al., 2015; Csonka et al., 2016; Sárközy et al., 2018). In contrast, experimental data are very limited to the connection between miRs and CKD-induced oxidative/nitrative stress in cardiac pathologies. Down-regulation of the anti-fibrotic miR-29 (Panizo et al., 2017; Drummond et al., 2018) and miR-30c (Panizo et al., 2017), as well as the up-regulation of the pro-fibrotic miR-21 (Chuppa et al., 2018), was observed in the heart of rats suffering from CKD. However, none of these studies investigated the link between the expressional change of miRs and cardiac oxidative/nitrative stress in CKD. In the future, miR-modulators might become new therapeutic approaches in T4CRS. Nevertheless, caution is needed with miR-modulators. Potentially harmful effects of miR-modulators could happen in non-targeted tissues (on-target side effects) or targeted tissues (off-target side effects including, e.g., chemical toxicity or unwanted gene expression changes). Therefore, the development of tissue-specific delivery techniques of miR-modulators is needed.

## Physical exercise and oxidative/nitrative stress in T4CRS in preclinical studies

Bai et al. demonstrated that voluntary running exercise for 4 weeks resulted in the development of LVH which was a functional adaptation in rats with CKD (Bai et al., 2009) (Table 4). In this study, exercise could ameliorate the upregulation of NOX-4 and NADPH oxidase subunits in the CKD rats (Bai et al., 2009) (Table 4). Another study showed that 4 weeks long voluntary wheel running preserved cardiac function, cardiac eNOS protein level, and NO bioavailability and decreased cardiac iNOS protein level in Sprague-Dawley rats underwent 5/6 nephrectomy (Kuczmarski et al., 2014) (Table 4). Chen et al. found that running or swimming reduced cardiac fibrosis in doxorubicin-induced CKD model. Both exercise regimes could increase the bioavailability of NO and the SOD level and decrease MDA level (Chen et al., 2014) (Table 4).

**Table 4:** Physical exercise and oxidative/nitrative stress in T4CRS in preclinical studies.

	**Experimental model**	**Age/body weight**	**FUP time**	**Characteristics of HF**	**Molecular markers of HF**	**Markers of cardiac oxidative/nitrative stress**	**Protective agent**	**References**
1	5/6 STNX female Sprague-Dawley rats	220–240 g	4 weeks	↑ atrial pressure	N/A	↑ NOX4, p22phox, gp91phox, Cu/Zn SOD, Mn SOD,	voluntary running exercise	Bai et al., 2009
2	5/6 ablation–infarction male Sprague-Dawley rats	12 weeks	8 weeks	LVH, systolic and diastolic dysfunction, ↓ cardiac work and rate pressure, ↓ response to left arterial filling and aortic pressure perturbation, ↓ cardiac output, ↓ stroke volume and stroke work post-ischemia,	N/A	↑ iNOS level, ↓ eNOS level, ↑ H2O2 production, ↓ SOD-1, ↑ GSH-Px1/2, CAT	voluntary wheel running	Kuczmarski et al., 2014
3	Doxorubicin-induced CKD male Sprague-Dawley rats	4 weeks	11 weeks	↑ HW/BW, ↓ capillary/fiber ratio, fibrosis	↑ cardiotrophin-1, collagen, IL-6; ↑ gp130, LIFR, p-STAT, p-JAK	↓ NO and SOD levels, ↑ MDA level	Treadmill running, swimming	Chen et al., 2014

*5/6 STNX, 5/6 subtotal nephrectomy; BW, body weight; CAT, catalase; CKD, chronic kidney disease; eNOS, endothelial nitric oxide synthase; FUP, follow-up; GSH-Px, glutathione peroxidase; gp130, glycoprotein 130; H_2_O_2_, hydrogen peroxide; HF, heart failure; HW, heart weight; IL-6, interleukin-6; iNOS, inducible nitric oxide synthase; LIFR, leukemia inhibitory factor receptor; LVH, left ventricular hypertrophy; MDA, malondialdehyde; NOX4, NADPH oxidase 4;; p-JAK, phospho-Janus kinase; p-STAT, signal transducer and activator of transcription; SOD, superoxide dismutase; Zn, zinc*.

## Physical exercise and oxidative/nitrative stress in T4CRS in clinical studies

In CKD patients, muscle catabolism and wasting are markedly common, and these result in decreased muscle strength, declines in physical function and activity (Wilund et al., 2010). Physical inactivity aggravates these functional changes and also triggers CVD (Wilund et al., 2010). Therefore, disability significantly reduces the quality of life and raises the mortality risk in ESRD patients. In a clinical pilot study enrolling 17 hemodialysis patients have been shown that intradialytic cycling exercise for 4 months increased performance on shuttle walking test and reduced the serum thiobarbituric acid reactive substances (TBARS), which is a marker of oxidative stress (Wilund et al., 2010). Moreover, exercise could reduce the thickness of the epicardial fat which is taught to be an inflammatory tissue secreting cytokines (Mazurek et al., 2003). It lies in close proximity to the adventitia of the coronary arteries and the myocardium suggesting that it might play a key role in the development of CVD (Mazurek et al., 2003).

## Pathways of oxidative/nitrative stress in renal sarcopenia

Not only systemic but also tissue oxidative/nitrative stress seems to be a major factor leading to renal sarcopenia. In contrast, there are only a limited number of studies which investigate skeletal muscle oxidative/nitrative stress and characterize both renal and skeletal muscle morphology and/or function in CKD. Therefore, in this section, we collected the studies which described both renal and muscle function and/or morphology and investigated the role of oxidative/nitrative stress in the development of renal sarcopenia.

## Skeletal muscle oxidative/nitrative stress in renal sarcopenia in preclinical studies

Experimental evidence showed that mainly the over-activation of NADPH-oxidase (Nishikawa et al., 2015; Enoki et al., 2016), especially its NOX4 isoform (Avin et al., 2016), the mitochondria (Gortan Cappellari et al., 2017), and iNOS (Chang et al., 2015; Oosterhuis et al., 2017) are the main sources of increased skeletal muscle ROS/RNS production in renal sarcopenia. Moreover, reduced bioavailability of NO due to reduced eNOS expression is also described in renal sarcopenia (Flisinski et al., 2012, 2017).

## Skeletal muscle oxidative/nitrative stress in renal sarcopenia in clinical studies

Crowe et al. investigated oxidative stress markers in skeletal muscle of ten hemodialysed patients and ten control subjects. Biopsies were taken from quadriceps femoris muscle and were analyzed for reduced and oxidized glutathione, SOD, catalase, protein thiols, MDA, and heat shock proteins (HSP27, HSP60, and HSP70) (Crowe et al., 2007). ESRD patients had muscle atrophy in both types I and II fibers, and reduced MDA content and catalase activity (Crowe et al., 2007). In contrast, total glutathione and heat shock protein levels were elevated, and other measured parameters were unchanged (Crowe et al., 2007). According to these findings, there is no clear link between oxidative stress and muscle fiber atrophy in ESRD; however, the small sample size is a limiting factor here. Lim et al. tested the hypothesis that the uremic condition may result in more damaged mitochondrial DNA (mtDNA) (Lim et al., 2000). In this study, multiple and diverse mtDNA deletions were found in the skeletal muscles of 22 ESRD patients (Lim et al., 2000). These mtDNA deletions were more common in ESRD patients than in normal populations. High levels of uremic toxins and impaired antioxidant mechanisms might be responsible for the elevated oxidative/nitrative stress in dialysis patients (Lim et al., 2000).

## Modulation of oxidative/nitrative stress in renal sarcopenia

To the best of our knowledge, there are no pharmaceutical or other approaches in the clinical practice to specifically target skeletal muscle oxidative/nitrative stress in renal sarcopenia. The most effective way for modulation of oxidative/nitrative stress in renal sarcopenia could be the prevention or treatment of CKD and its cardiovascular complications because T4CRS and renal sarcopenia could worsen each other. Well-known and clinically used drugs for the therapy of the cardiovascular complications of CKD might have antioxidant properties or reduce the cardiovascular risk in CKD (e.g., RAAS inhibitors) (see section Modulation of Oxidative/Nitrative Stress in T4CRS) which might be a therapeutic option. However, the number of studies is very limited on drugs which directly or indirectly target skeletal muscle oxidative/nitrative stress in renal sarcopenia. In this section, we collected both experimental and clinical studies which examined the effects of various pharmaceuticals on CKD-induced oxidative/nitrative stress in renal sarcopenia.

## Pharmaceuticals modulating skeletal muscle oxidative/nitrative stress in renal sarcopenia in preclinical studies

### Unacylated ghrelin

Ghrelin is a hormone produced by the gastrointestinal tract and regulates food intake and energy homeostasis (Yanagi et al., 2018). Acylated ghrelin induces hyperglycemia and reduces the insulin secretion, and insulin sensitivity (Yanagi et al., 2018). In contrast, unacylated ghrelin antagonizes these effects (Yanagi et al., 2018). Incubation of C2C12 myotubes with human uremic serum resulted in higher mitochondrial ROS generation which could be ameliorated by unacylated ghrelin (Gortan Cappellari et al., 2017) (Table 5). In this study, 4-days treatment with unacylated ghrelin (200 mg, s.c. ghrelin injection twice a day) enhanced mitophagy, normalized oxidative stress, inflammation, and impaired insulin signaling as well as muscle loss in the gastrocnemius muscle in 5/6 nephrectomized rats (Gortan Cappellari et al., 2017) (Table 5).

**Table 5:** Pharmaceuticals modulating skeletal muscle oxidative/nitrative stress in renal sarcopenia in preclinical studies.

	**Experimental model**	**Age/body weight**	**FUP time**	**Characteristics of HF**	**Molecular markers of HF**	**Markers of skeletal muscle oxidative/nitrative stress**	**Protective agent**	**References**
	Uremic or control human serum treated C2C12 myoblasts	N/A	4–7 days	N/A	N/A	High mitochondrial superoxide production	Unacetylated ghrelin (UnaG)	Gortan Cappellari et al., 2017
1	5/6 STNX male Wistar rats (M. gastrocnemius)	12 weeks	40 days	↓ body weight gaining, ↓ muscle mass	↑ muscle proteolysis and ↑ actin fragmentation	↑ mitochondrial superoxide and H2O2 production, ↑ oxidized/total GSH ratio, ↑ ROS production	Unacetylated ghrelin
	C2C12 myoblast	N/A	1 day	N/A	N/A	↑ NADPH oxidase activity	-	Nishikawa et al., 2015
2	5/6 STNX male C57BL/6J mice (M. Gastrocnemius, M. Quadriceps, M. Soleus)	6–7 week-old	21 weeks	↓ cell cross-sectional area, ↓ mitochondrial biogenesis and density, ↓ running distance and run time to exhaustion	CD31 staining for angiogenesis	↑ superoxide production, ↑ NADPH oxidase subunits expression and activity	AST-120 -oral absorbent

*5/6 STNX, 5/6 subtotal nephrectomy; FUP, follow-up; GSH, glutathione; H_2_O_2_, hydrogen peroxide*.

### Oral charcoal adsorbent AST-120

The oral adsorbent AST-120 is thought to be able to decrease the levels of IS (Nishikawa et al., 2015) (Tables 4, 5). Nishikawa et al. found that AST-120-enriched diet for 8 weeks could reduce plasma IS levels and superoxide production in the skeletal muscle in 5/6 nephrectomized mice (Nishikawa et al., 2015) (Tables 4, 5). In this model of CKD, the AST-120 treatment resulted in better running distances to exhaustion, which was determined by treadmill tests (Nishikawa et al., 2015) (Table 5).

## Pharmaceuticals modulating skletal muscle oxidative/nitrative stress in renal sarcopenia in clinical studies

### Creatine supplementation

ESRD patients undergoing chronic dialysis have severe energy depletion caused by the decreased phosphoryl-creatine/ATP ratio in skeletal muscle (Wallimann et al., 2017). Wallimann et al. summarized in a review that intradialytic creatine supplementation could prevent mitochondria from oxidative stress by reducing the production of ROS (Wallimann et al., 2017).

## Novel approaches modulating skeletal muscle oxidative/nitrative stress in renal sarcopenia in preclinical studies

### microRNAs (miRs)

Multiple miRs (e.g., miR-133, miR-23, miR-26, miR-27, miR-214, miR-182, miR-221/222, miR-486, etc.) are implicated in muscle cell differentiation and muscle mass regulation in renal sarcopenia targeting the elements of the IGF1, myostatin and GH-mediated pathways (Mak and Cheung, 2012; Hudson et al., 2014; Wang et al., 2017). However, it is unclear whether these or other miRs are regulated by oxidative/nitrative stress in renal sarcopenia. Further studies are needed for the investigation of the direct link between skeletal muscle oxidative/nitrative stress and expression changes of miRs in renal sarcopenia. In the future, miR modulators might represent new therapeutic approaches for muscle wasting also in renal sarcopenia. Nevertheless, miR targets need to be specific to skeletal muscle, since undesirable side effects may develop in other organs.

## Conclusions and perspectives

In CKD, cardiac and skeletal muscle injury develops as a consequence of a myriad of maladaptive mechanisms including systemic inflammation and neurohormonal activation which could be over-activated and perpetuated by the increased oxidative/nitrative stress. These mechanisms could participate in the complex interorgan crosstalk between the kidneys, heart and skeletal muscle. Moreover, pathological states of one or more of the aforementioned organs can lead to morphological and functional changes in other organs. Despite the accumulating evidence based mainly on preclinical studies, there is no breakthrough in the treatment of uremic cardiomyopathy. Its treatment remains largely symptomatic by well-known pharmaceuticals used for the treatment in other forms of heart failure (e.g., RAAS inhibitors). Elevated oxidative/nitrative stress and inflammation are key elements in the complex pathomechanism and interrelationship between T4CRS and renal sarcopenia.

**Figure 4:**
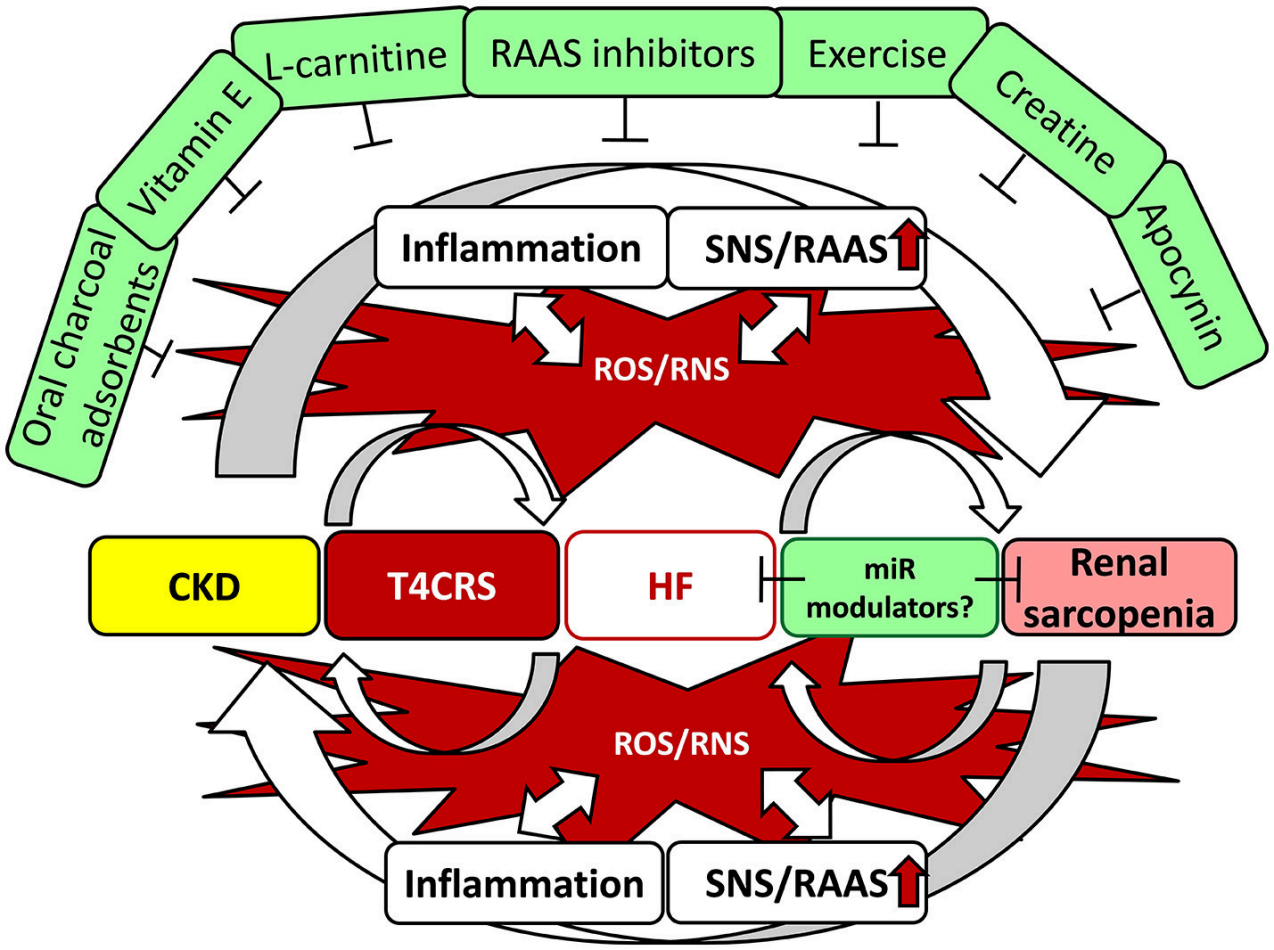
Interrelationship between T4CRS and sarcopenia. CKD, chronic kidney disease; CRS, cardio-renal syndrome; HF, heart failure; miR, microRNA; RNS, reactive nitrogen species; RAAS, renin–angiotensin–aldosterone system; ROS, reactive oxygen species; SNS, sympathetic nervous system.

In contrast, there are only a limited number of clinical studies which targets indirectly or directly CKD-induced oxidative/nitrative stress, and their results are largely disappointing. Therefore, the thorough understanding of the mechanisms which lead to perturbations in oxidative/nitrative metabolism and its relationship with pro-inflammatory, hypertrophic, fibrotic, cell death and other pathways would help to develop strategies to counteract systemic and tissue-specific oxidative/nitrative stress to control the progression of CKD and the end-organ damages in the heart and skeletal muscle (Figure 4). Moreover, many of the possible modulators of oxidative/nitrative stress (e.g., antioxidants, nutraceuticals, exercise, etc.) require further optimization for application in CKD patients. Novel therapies (e.g., miR modulators, selective inhibitors, etc.) which target NADPH oxidase, uncoupled eNOS, iNOS or mitochondrial ROS generation are also needed.

## Author contributions

MS, ZK, MK, RG, and GS searched literature. MS, ZK, MK, and GS drafted manuscript. RG performed Tables, MS performed Figures. MS and LD edited and revised the manuscript. All authors read and approved the final version of the manuscript.

### Conflict of interest statement

The authors declare that the research was conducted in the absence of any commercial or financial relationships that could be construed as a potential conflict of interest.

## Abbreviations

AGEs: advanced glycation end products
AT1: angiotensin II receptor type 1
ATP: adenosine triphosphate
CAT: catalase
CKD: chronic kidney disease
CVD: cardiovascular diseases
CoQ10: coenzyme Q10
COX: cyclooxygenase
CRP: C-reactive protein
DNA: deoxyribonucleic acid
eGFR: estimated glomerular filtration rate
eNOS: endothelial nitric oxide synthase
ER: endoplasmic reticulum
ERK-1/2: extracellular signal-regulated protein kinases 1 and 2
ESRD: end stage renal disease
FGF: fibroblast growth factor
GFR: glomerular filtration rate
GSH: glutathione
GSH-Px: glutathione peroxidase
GST: glutathione S-transferase
H_2_O_2_: hydrogen peroxide
HDL: high density lipoprotein
HFpEF: heart failure with preserved ejection fraction
HFrEF: heart failure with reduced ejection fraction
ICAM: intercellular adhesion molecule 1
IL: interleukin
iNOS: inducible nitric oxide synthase
JNK: c-Jun N-terminal kinase
LDL: low density lipoprotein
LVH: left ventricular hypertrophy
MAPK: mitogen-activated protein kinase
MDA: malondialdehyde
MPO: myeloperoxidase
NADPH oxidase: nicotinamide adenine dinucleotide phosphate oxidase
NF-κB: nuclear factor kappa-light-chain-enhancer of activated B cells
^·^NO: nitric oxide
nNOS: neuronal nitric oxide synthase
NOXs: NADPH oxidases
Nrf2: nuclear factor erythroid 2–related factor 2
NT-pro-BNP: N-terminal pro-brain natriuretic peptide
${\text{O}}_{2}^{\cdot -}$: superoxide
^·^OH: hydroxyl radical
ONOO^−^: peroxynitrite
PARP-1: poly-ADP-ribose-polymerase 1
PCS: p-cresyl sulfate
PDGF: platelet-derived growth factor
PON-1: paraoxonase/arylesterase 1
RAAS: renin–angiotensin–aldosterone system
RNS: reactive nitrogen species
ROS: reactive oxygen species
SERCA2a: sarcoplasmic reticulum calcium ATPase 2a
SNS: sympathetic nervous system
SOD: superoxide dismutase
T4CRS: type 4 cardio-renal syndrome
TBARS: thiobarbituric acid reactive substances
TGF-β: transforming growth factor-beta
TNF-α: tumor necrosis factor-alpha
VCAM: vascular cell adhesion molecule 1
VEGF: vascular endothelial growth factor
